# Calcium Mobilization And Protein Kinase C Activation Downstream Of Protease Activated Receptor 4 (PAR4) Is Negatively Regulated By PAR3 In Mouse Platelets

**DOI:** 10.1371/journal.pone.0055740

**Published:** 2013-02-06

**Authors:** Amal Arachiche, María de la Fuente, Marvin T. Nieman

**Affiliations:** Department of Pharmacology, Case Western Reserve University, Cleveland, Ohio, United States of America; Emory University/Georgia Insititute of Technology, United States of America

## Abstract

Thrombin activates platelets through protease activated receptors (PARs). Mouse platelets express PAR3 and PAR4. PAR3 does not signal in platelets. However, PAR4 is a relatively poor thrombin substrate and requires PAR3 as a cofactor at low thrombin concentrations. In this study we show that PAR3 also regulates PAR4 signaling. In response to thrombin (30–100 nM) or PAR4 activating peptide (AYPGKF), platelets from PAR3^−/−^ mice had increased G_q_ signaling compared to wild type mice as demonstrated by a 1.6-fold increase in the maximum intracellular calcium (Ca^2+^) mobilization, an increase in phosphorylation level of protein kinase C (PKC) substrates, and a 2-fold increase of Ca^2+^ release from intracellular stores. Moreover, platelets from heterozygous mice (PAR3^+/−^) had an intermediate increase in maximum Ca^2+^ mobilization. Treatment of PAR3^−/−^ mice platelets with P2Y_12_ antagonist (2MeSAMP) did not affect Ca^2+^ mobilization from PAR4 in response to thrombin or AYPGKF. The activation of RhoA-GTP downstream G_12/13_ signaling in response to thrombin was not significantly different between wild type and PAR3^−/−^ mice. Since PAR3 influenced PAR4 signaling independent of agonist, we examined the direct interaction between PAR3 and PAR4 with bioluminescence resonance energy transfer (BRET). PAR3 and PAR4 form constitutive homodimers and heterodimers. In summary, our results demonstrate that in addition to enhancing PAR4 activation at low thrombin concentrations, PAR3 negatively regulates PAR4-mediated maximum Ca^2+^ mobilization and PKC activation in mouse platelets by physical interaction.

## Introduction

Thrombin signaling in platelets is mediated by protease activated receptors (PARs). PARs are G-protein-coupled receptors (GPCR) that are activated via proteolytic cleavage of the extracellular N-terminus to initiate a variety of signaling cascades through activation of heterotrimeric G proteins [1], [2]. The expression of PARs in platelets is species specific. Human platelets express PAR1 and PAR4 and cleavage of each receptor initiates signaling cascades [3]. Mouse platelets express PAR3 and PAR4, but PAR3 does not signal making PAR4 the signaling receptor [4]–[6].

PAR1 and PAR4 in human platelets are coupled to G_q_ and G_12/13_ [7], [8]. The presence of a direct interaction between PARs and G_i_ is controversial. It has been shown that PAR1 is directly coupled to G_i_ in human platelets and in COS7 cells transfected with PAR1 [9], [10]. However, other studies have shown that PAR1 and PAR4 do not couple directly to G_i_, rather activation of the G_i_ pathway is mediated by secondary release of ADP, which acts on the G_i_-coupled ADP receptor, P2Y_12_ [8], [11], [12].

A common feature of PAR4 across species is that, on its own, PAR4 is not an efficient thrombin substrate [13]–[15]. As a result, PAR1 in human platelets or PAR3 in mouse platelets serves as a cofactor for PAR4 activation at low thrombin concentrations (<10 nM). However, at high concentrations of thrombin (≥30 nM), PAR4 is sufficient to induce platelet activation [6]. Two independent studies show that PAR3 can affect PAR4 signaling, Nakanishi-Matsui *et al*, reported that the amount of accumulated inositol phosphate (IP) in response to thrombin (10–100 nM) was 1.7-fold increased in COS7 cells expressing mouse PAR4 alone compared to COS7 cells expressing mouse PAR4 and PAR3 [6]. In addition, Mao *et al*. showed an increase in intracellular Ca^2+^ mobilization and platelet aggregation in response to plasmin, in PAR3 knockout (PAR3^−/−^) mouse platelets compared to wild type [16]. These studies show that PAR3 can influence PAR4 signaling in addition to enhancing PAR4 activation. There are also examples of PAR3 regulating signaling from other PAR family members in endothelial cells and podocytes [17], [18].

In the present study we aimed to determine if the activation of PAR4 with thrombin concentrations that occur at the site of the growing thrombus [19] is affected by the presence of PAR3 in mouse platelets. We report here that PAR3 negatively regulates PAR4-mediated G_q_ signaling by down regulation of Ca^2+^ mobilization and PKC activation without affecting the G_12/13_ pathway as measured by RhoA activation. The negative regulation of PAR3 on PAR4 signaling was independent of the PAR4 agonist. Therefore, we examined the physical interaction between PAR3 and PAR4 with bioluminescence resonance energy transfer (BRET). We also show for the first time that PAR3 forms a constitutive heterodimer with PAR4, and this interaction may affect PAR4 signaling when PAR3 is absent. The results from this study demonstrate that PAR4 signaling can be modulated by other PAR subtypes at thrombin concentrations that are found *in vivo* at the site of the thrombus. This may have important implications for PAR4 signaling in human platelets where it is co-expressed with PAR1. More generally, the physical interaction between platelet GPCRs may provide unique signaling and may have broad implications for the design of antiplatelet agents.

## Materials and Methods

### Reagents and Antibodies

Human α-thrombin (specific activity of 5380 NIH units/mg) was purchased from Haematological Technologies (Essex Junction, VT). PAR4 activating peptide (AYPGKF-NH2) was synthesized at PolyPeptide Laboratories (San Diego, CA). Convulxin was purchased from Enzo Life Sciences Inc. (Farmingdale, NY). ADP was purchased from Chrono-log Corporation (Havertown, PA). Fura-2AM and all cell culture reagents were purchased from Invitrogen. Prostaglandin I_2_ was purchased from Calbiochem. Heparin, thapsigargin, and 2-Methylthioadenosine 5′-monophosphate triethylammonium salt hydrate (2MeSAMP) were purchased from Sigma Chemical Co. The anti-phospho-(Ser) PKC substrates, anti-PKC, anti-phospho-Akt (Ser473) antibodies were purchased from Cell Signaling Technology Inc. (Danvers, MA). The anti-α-actinin antibody was purchased from Santa Cruz Biotechnology Inc. (Santa Cruz, CA). The anti-PAR4-FITC antibody was purchased from Alamone Labs Ltd. (Jerusalem, Israel). The anti-HA tag Alexa Fluor 647 (6E2) antibody was purchased from Cell Signaling Technology Inc. (Danvers, MA). The anti-V5 tag Alexa Fluor 647 antibody was purchased from AbD Serotec. (Raleigh, NC).

### Animals

PAR3 knockout (PAR3^−/−^) and PAR3 heterozygous (PAR3^+/−^) mice have been described and were obtained from Mutant Mouse Regional Resource Center (MMRRC, Chapel Hill, NC) [5]. All animal studies were approved by the Institutional Animal Care and Use Committee at Case Western Reserve University School of Medicine.

### Platelet preparation

Mice were anesthetized with intraperitoneal injection of pentobarbital (62 mg/kg). Blood was collected from mice by heparinized capillary puncture of the retro-orbital venous sinus and immediately combined with (1/5) volume of acid citrate dextrose (ACD) as an anticoagulant. The whole blood was centrifuged at 2300× *g* for 20 sec at room temperature (RT) to isolate platelet-rich plasma (PRP). The platelets were pelleted and washed once at 2200× *g* for 3 min at RT in HEPES-Tyrode buffer pH 7.4 (10 mM HEPES, 12 mM NaHCO_3_, 130 mM NaCl, 5 mM KCl, 0.4 mM Na_2_HPO_4_, 1 mM MgCl_2_, 5 mM glucose, 0.33% w/v human serum albumin) containing 0.5 µM prostaglandin I_2_ (PGI_2_) and 10 U/mL Heparin. Washed platelets were counted on a Hemavet 950FS (Drew Scientific Inc, Waterbury, CT, USA) and the final platelet count adjusted with HEPES-Tyrode's buffer.

### Measurement of the concentration of free intracellular Ca^2+^ ([Ca^2+^]_i_)

Washed mouse platelets adjusted to a final concentration of 2×10^8^ platelets/mL were loaded with 10 µM Fura-2 for 45 minutes at room temperature. Platelets were washed once and resuspended to their original concentration in HEPES-Tyrode buffer (pH 7.4) containing 2 mM CaCl_2_ or 0.1 mM EGTA. In some experiments, Fura-2 loaded platelets were treated with 100 µM 2-MeSAMP for 5 min in the dark at 37°C prior to measuring intracellular Ca^2+^ mobilization. Ca^2+^ release from internal stores was determined by stimulating platelets with 3 µM thapsigargin. Eighty microliters of Fura-2 loaded platelets were placed in 96-well plates, stimulated with agonist, and read in a NOVOstar plate reader (BMG Labtech, Durham, NC) at 37°C. Intracellular Ca^2+^ variations were monitored by measuring the Fura-2 fluorescence ratio at 340/380 nm with emission at 510 nm. Fluorescence measurement was converted to the concentration of intracellular free Ca^2+^ using equation reported by Grynkiewicz *et al*. [20].

### Measurement of PAR4 expression in mouse platelets

Mouse platelet surface expression of PAR4 was determined by flow cytometry using a Beckman Coulter LSRII (Case Comprehensive Cancer Center Flow Core). Washed mouse platelets were adjusted to a final concentration of 40×10^6^ platelets/mL in HEPES-Tyrode buffer (pH 7.4). Twenty-five microliter aliquots were incubated with 25 µg/mL of anti-PAR4-FITC at 4°C for 20 min. Platelets were then diluted (1∶8) and 10,000 events were acquired on a Beckman Coulter LSRII. Flow cytometry data were analyzed with Flowjo software.

### Western blotting

Washed platelets were adjusted to a final concentration of 3×10^8^ platelets/mL. Fifty microliter aliquots were activated with thrombin(1, 10, 30, or 100 nM) or AYPGKF (0.03, 0.5, 1.5, or 2 mM) for 3 min at 37°C. The reaction was stopped by adding 6× Laemmli reducing buffer and samples were resolved by SDS-PAGE and transferred onto polyvinylidene difluoride (PVDF) membranes. Membranes were incubated with primary rabbit antibody to phospho-(Ser) PKC substrates, PKC, or phospho-Akt (Ser473). To demonstrate protein loading, the membrane was reprobed with a rabbit antibody to α-actinin. Detection was performed with HRP-conjugated anti-rabbit secondary antibody and an enhanced chemiluminescence system (Pierce Chemical). The optical densities of proteins in the blot were quantified using Image J (1.45) software.

### ELISA-based RhoA activation assay

RhoA GTPase activity was determined in freshly prepared mouse platelets lysates (adjusted to a final concentration of 3×10^8^ platelets/mL) by using the absorbance based G-LISA RhoA activation assay kit (Cytoskeleton, Inc.). Fifty microliter aliquots were activated with thrombin (1, 10, 30, or 100 nM) for 3 min at 37°C. Activated mouse platelets were then lysed using an equal volume of supplied cell lysis buffer, and the lysates were immediately used for the Rho G-LISA assay. All subsequent incubation and detection followed the instructions provided by the manufacturer.

### Bioluminescence resonance energy transfer (BRET) assay and cell surface expression of mouse PAR3 and mouse PAR4 measurement

The cDNA for mouse PAR3 was purchased from Origene Technologies Inc. (Rockville, MD) and the cDNA for mouse PAR4 was isolated from a BaF3 cDNA library. An amino terminal HA (YPYDVPDYA) or V5 (GKPIPMPLLGLDST) epitope tag was added to PAR3 or PAR4 by PCR, and the cDNAs were ligated into pLUC-N2 (HA-tagged) or pGFP^2^-N2 (V5 tagged). The mouse rhodopsin in pGFP^2^-N2 (V5 tagged) has been described [21] . The BRET vectors were purchased from PerkinElmer. All constructs were verified by DNA sequencing. For BRET assays, HEK293 cells (American Type Culture Collection) were transfected with Lipofectamine 2000 according to manufacturer's instructions. BRET assays were performed as previously described [21].

Cell surface expression of mouse PAR3 and mouse PAR4 was determined by flow cytometry using BD LSRFortessa (Center for Aids Research, Immune Function Core, Case Western Reserve University). HEK293 cells (1×10^5^) in 6-well plates were transfected with 1.5 µg of HA-PAR4-LUC or V5-PAR4-GFP and 1 µg of HA-PAR3-LUC or V5-PAR3-GFP. Transfected cells were removed from plates 48 h post-transfection by rinsing with PBS. Surface detection of HA- tagged PAR3 or PAR4 was detected with an HA tag antibody conjugated to Alexa Fluor 647 (Cell Signaling Technology Inc) with 1∶50 dilution. Surface detection of V5- tagged PAR3 or PAR4 was detected with a V5 tag antibody conjugated to Alexa Fluor 647 (AbD Serotec) with 1∶5 dilution. HEK293 labeled cells (1.25×10^5^) were then diluted (1∶8) and 10,000 events were acquired on a BD LSRFortessa. Cell surface expression of HA- or V5-tagged PAR4 or PAR3 was determined by quantitative flow cytometry and performed essentially as described [21].

### Data analysis

Differences between means were determined by unpaired Student's *t* test and by one way ANOVA test and were considered significant when *p*<0.05.

## Results

### Intracellular Ca^2+^ mobilization is increased in PAR3^−/−^ mouse platelets

**Figure 1:**
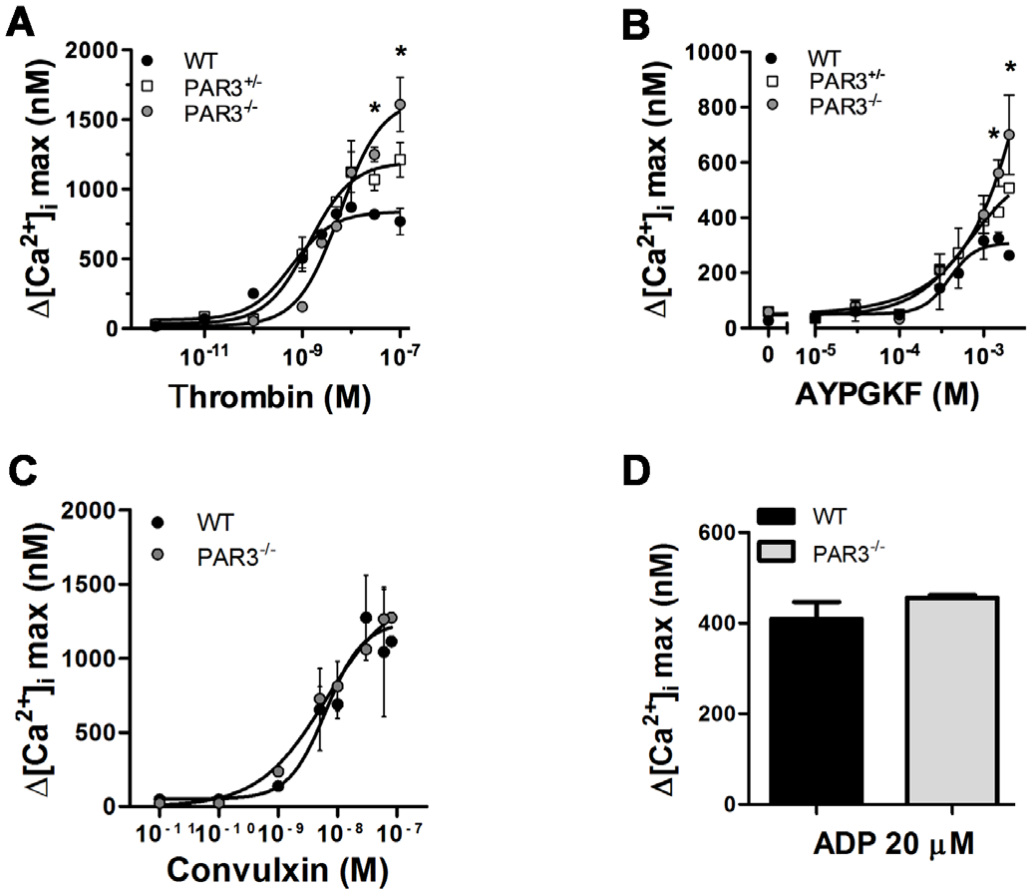
Dose response curve of Ca^2+^ mobilization in the presence of extracellular Ca^2+^ in mouse platelets. Fura 2-loaded wild type (black circle), PAR3^−/−^ (gray circle), and PAR3^+/−^ (white square) platelets were activated with the indicated concentrations of: (**A**) thrombin, (0.001–100 nM, (**B**) AYPGKF (0–2 mM), (**C**) convulxin (0.01–100 nM), or 20 µM of ADP for 10 min at 37°C in the presence of 2 mM of CaCl_2_. The difference between the maximum increase and the basal intracellular Ca^2+^ mobilization was measured. The results are the mean (± SD) of three independent experiments (* *p*<0.05).

**Figure 2:**
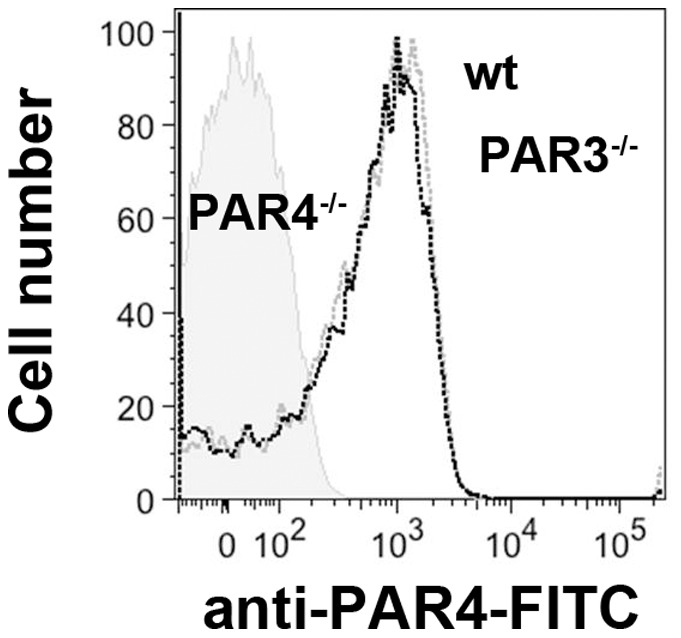
PAR4 expression on mouse platelets. Flow cytometric analysis of PAR4 expression in wild type (WT) (black line), PAR3^−/−^ (gray line), and PAR4^−/−^ (shaded) mice platelets using anti-PAR4-FITC antibodies.

We first determined if the absence of PAR3 affected PAR4 mediated intracellular Ca^2+^ mobilization in PAR3^−/−^ platelets in response to thrombin. The EC_50_ for thrombin-induced Ca^2+^ mobilization is increased ∼10-fold in PAR3^−/−^ platelets compared to wild type platelets (4.1 nM vs 0.6 nM, with a 95% confidence interval of 0.24–1.5 nM or 2.3–15 nM, respectively) (Figure 1A). Heterozygous mice (PAR3^+/−^) had an intermediate value (1.1 nM with a 95% confidence interval of 0.5–3.7 nM). These results agree with published data showing that PAR3 is a cofactor for PAR4 activation at low thrombin concentrations [6]. However, at thrombin concentrations above 10 nM, platelets from PAR3^−/−^ mice had a ∼1.6-fold increase in the maximum Ca^2+^ mobilization compared to wild type platelets. Platelets from PAR3^+/−^ had an intermediate increase in the maximum Ca^2+^ mobilization (∼1.2-fold) (Figure 1A). These data indicated that the absence of PAR3 affects the Ca^2+^ mobilization in response to high thrombin concentrations (30–100 nM). We next determined if the increase in the maximum Ca^2+^ mobilization in PAR3^−/−^ platelets was dependent on thrombin's interaction with PAR4 by using a specific PAR4 activating peptide (AYPGKF). Similar to thrombin treatment, platelets from PAR3^−/−^ and PAR3^+/−^ mice had a 2.6-fold or 1.9-fold increase in the maximum Ca^2+^ mobilization, respectively, compared to wild type platelets in response to AYPGKF (Figure 1B). The EC_50_ for AYPGKF-induced Ca^2+^ mobilization is not statistically significant between wild type and PAR3^−/−^ platelets (392 µM vs. 614 µM, respectively, *p = *0.45). Next, we investigated whether the increase in the maximum Ca^2+^ mobilization in the PAR3^−/−^ mice was specific to PAR4 stimulation. Wild type and PAR3^−/−^ platelets were stimulated with convulxin, the specific GPVI agonist, or with a high concentration of ADP [22], the specific P2Y_12_ and P2Y_1_ agonist. There was no significant difference in the maximum Ca^2+^ mobilization or the EC_50_ in response to convulxin (Figure 1C) or in the maximum Ca^2+^ mobilization in response to 20 µM ADP (Figure 1D) between wild type and PAR3^−/−^ platelets. These data indicate that the increase in the maximum Ca^2+^ mobilization was specific to PAR activation, but independent of the PAR4 agonist. These data suggest that PAR3 influences PAR4 at the level of the receptor. To verify that the increase in the maximal Ca^2+^ mobilization was not due to an increase in surface expression of PAR4 in PAR3^−/−^ platelets, PAR4 expression was measured by flow cytometry. Platelets from wild type and PAR3^−/−^ mice had the same level of PAR4 expression (Figure 2).

### P2Y_12_ inhibition does not influence PAR4 enhanced Ca^2+^ mobilization in PAR3^−/−^ mouse platelets

**Figure 3:**
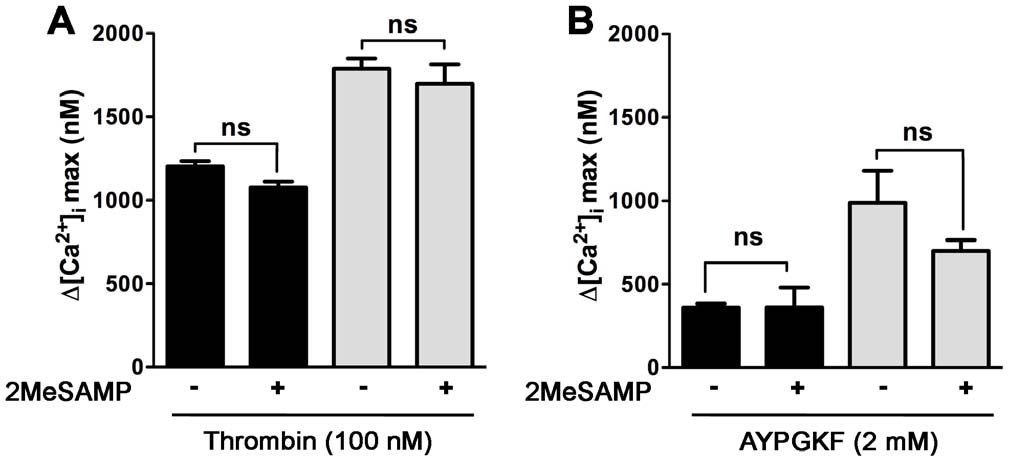
Effect of 2MeSAMP on PAR4 enhancing intracellular Ca^2+^ mobilization in mouse platelets. Fura 2-loaded wild type (black) and PAR3^−/−^ (gray) platelets were incubated at 37°C for 5 min in the absence or the presence of 100 µM 2MeSAMP. After treatment, platelets were activated 100 nM thrombin (**A**,) or 2 mM AYPGKF (**B**) for 10 min at 37°C in the presence of 2 mM of CaCl_2_. The difference between the maximum increase and the basal intracellular Ca^2+^ mobilization was measured. The results are the mean (± SD) of three independent experiments (* *p*<0.05).

PAR4 and P2Y_12_ physically interact in human platelets after thrombin or AYPGKF stimulation and the association is reduced by P2Y_12_ inhibitor 2MeSAMP [23]. To determine if the increase in the maximum Ca^2+^ mobilization was caused by crosstalk between PAR4 and P2Y_12_ in the absence of PAR3, wild type and PAR3^−/−^ platelets were stimulated with thrombin or AYPGKF in the presence of 2MeSAMP (P2Y_12_ antagonist). There was no significant difference in the maximum Ca^2+^ mobilization between wild type and PAR3^−/−^ platelets activated with 30 nM thrombin (*p = *0.64, data not shown) or 100 nM thrombin (*p = *0.99, Figure 3A). Similarly, there was no significant difference in maximum Ca^2+^ mobilization when platelets were stimulated with 1.5 mM AYPGKF (*p = *0.10, data not shown) or 2 mM AYPGKF (*p = *0.06, Figure 3B). These data indicate that the increase in the maximum Ca^2+^ mobilization was independent of the PAR4-P2Y_12_ interaction after thrombin or AYPGKF stimulation.

### Protein Kinase C (PKC) activation is increased in PAR3^−/−^ mouse platelets

**Figure 4:**
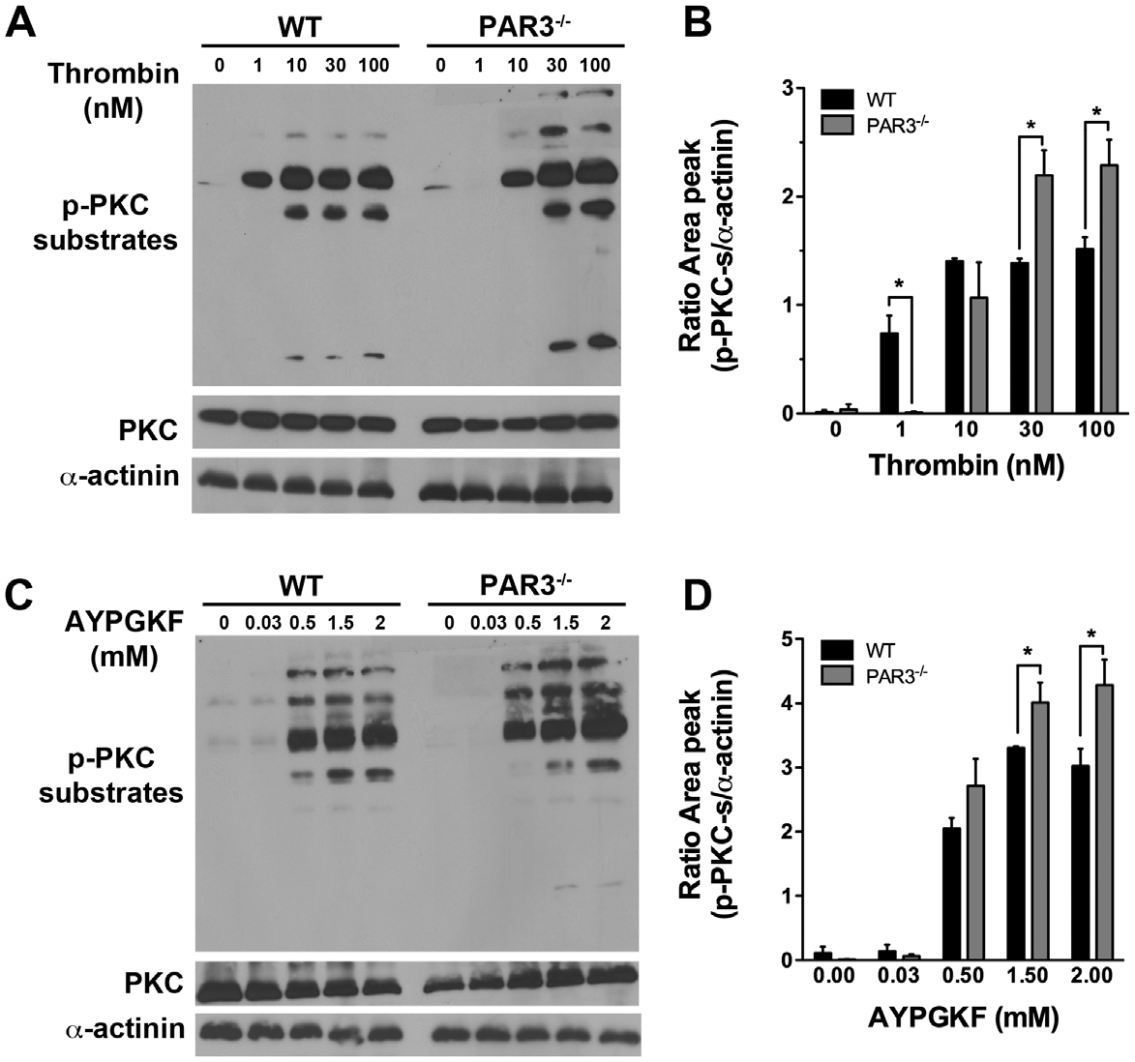
Western blot analysis of protein kinase C (PKC) substrate phosphorylation in mouse platelets. The level of PKC substrate phosphorylation on serine residues in response to increasing concentrations of: (**A**) thrombin (1–100 nM) or (**C**) AYPGKF (0.03–2 mM) was determined by western blotting with phospho-(Ser) PKC substrate antibody. The membranes were re-probed for α-actinin to demonstrate protein loading. The blots shown are from a representative of three independent experiments. Quantitation of PKC substrate phosphorylation in response to (**B**) thrombin or (**D**) AYPGKF is represented at the mean (± SD) (* *p*<0.05).

Intracellular Ca^2+^ mobilization and PKC activation are both downstream of G_q_. We next determined if PKC activation was also increased in PAR3^−/−^ platelets by measuring the serine phosphorylation of PKC substrates, which reflects the activation of PKC. Serine phosphorylation on PKC substrates in response to 1 nM thrombin was absent in PAR3^−/−^ platelets compared to wild type platelets (Figure 4A and B). These results are expected because PAR3 is required for PAR4 activation at low concentrations of thrombin. Importantly, we show that the level of serine phosphorylation of PKC substrates was increased in response to high concentration of thrombin (30–100 nM) in PAR3^−/−^ compared to wild type mouse platelets. The level of serine phosphorylation on PKC substrates was also increased in response to AYPGKF in PAR3^−/−^ platelets (Figure 4C and D). To show that the increased PKC activity in PAR3^−/−^ platelets was not due to increased expression of PKC, we reprobed the membranes for total PKC. There was no significant difference in the level of PKC between PAR3^−/−^ and wild type platelets. These results are consistent with the Ca^2+^ mobilization data and indicate that PAR3 negatively regulates PAR4-induced G_q_-mediated signaling in mouse platelets.

### Intracellular Ca^2+^ store depletion is increased in PAR3^−/−^ mouse platelets

**Figure 5:**
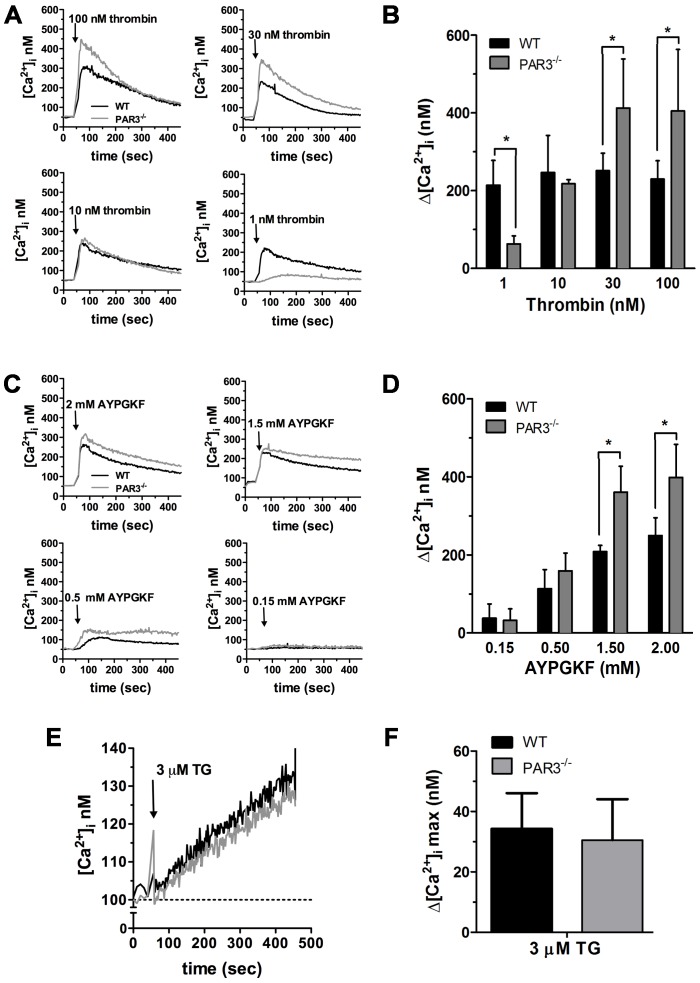
Dose response curve of Ca^2+^ mobilization in the absence of extracellular Ca^2+^ in mouse platelets. Fura 2-loaded wild type (black line) and PAR3^−/−^ (gray line) platelets were resuspended in Ca^2+^-free medium (0.1 mM EGTA was added at the time of experiment). Representative tracings are shown from platelets activated with the indicated concentrations of: (**A**) thrombin (1–100 nM), (**C**) AYPGKF (0.15–2 mM), or (**E**) 3 µM thapsigargin (TG). Quantitation of the change in peak Ca^2+^ mobilization in platelets stimulated with: (**B**) thrombin, (**D**) AYPGKF, or (**F**) thapsigargin. The results are the mean (± SD) of three independent experiments (* *p*<0.05).

We next examined if the increase in the maximum Ca^2+^ mobilization was caused by an increase in the depletion of intracellular Ca^2+^ stores. Platelets from PAR3^−/−^ and wild type mice were stimulated with thrombin (1, 10, 30, or 100 nM) in Ca^2+^-free buffer (0.1 mM EGTA added). At 1 nM thrombin, the depletion of intracellular Ca^2+^ stores was decreased in PAR3^−/−^ compared to wild type platelets. These data are consistent with PAR3 facilitating PAR4 activation at low thrombin concentrations. However, at thrombin concentrations above 10 nM, platelets from PAR3^−/−^ release more Ca^2+^ from the internal stores compared to wild type platelets (Figure 5A and B). The maximum Ca^2+^ mobilization from the intracellular stores in PAR3^−/−^ platelets was 405±158 nM compared to 229±47 nM for wild type platelets, *p* = 0.04 (Figure 5B). Similar to thrombin stimulation, PAR3^−/−^ platelets release more Ca^2+^ from their internal stores compared to wild type platelets in response to AYPGKF (Figure 5C and D). However, in response to 3 µM thapsigargin, there is no difference in Ca^2+^ release from the internal stores between PAR3^−/−^ and wild type platelets (Figure 5E and F). These data indicate that PAR3^−/−^ has the same Ca^2+^ pool in the internal stores compared to wild type platelets. However, after activation, there is more Ca^2+^ released from the intracellular stores for PAR3^−/−^ platelets compared to wild type platelets.

### G_12/13_ and G_i_-mediated signaling are not affected in PAR3^−/−^ mouse platelets

**Figure 6:**
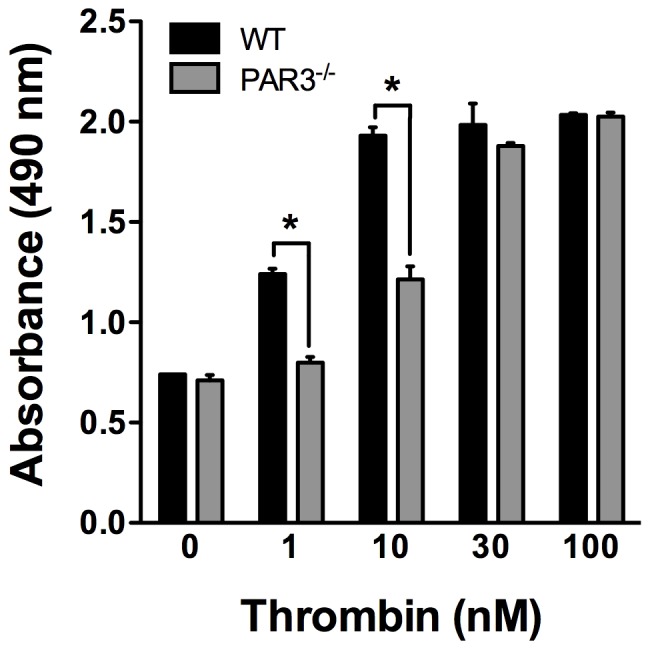
RhoA activity measured by G-LISA Kit in mouse platelets. The level of activated RhoA-GTP is measured by absorbance at 490 nm in response to increasing concentrations of thrombin (1–100 nM). The results are from three independent experiments (* *p*<0.05, ns: not significant).

**Figure 7:**
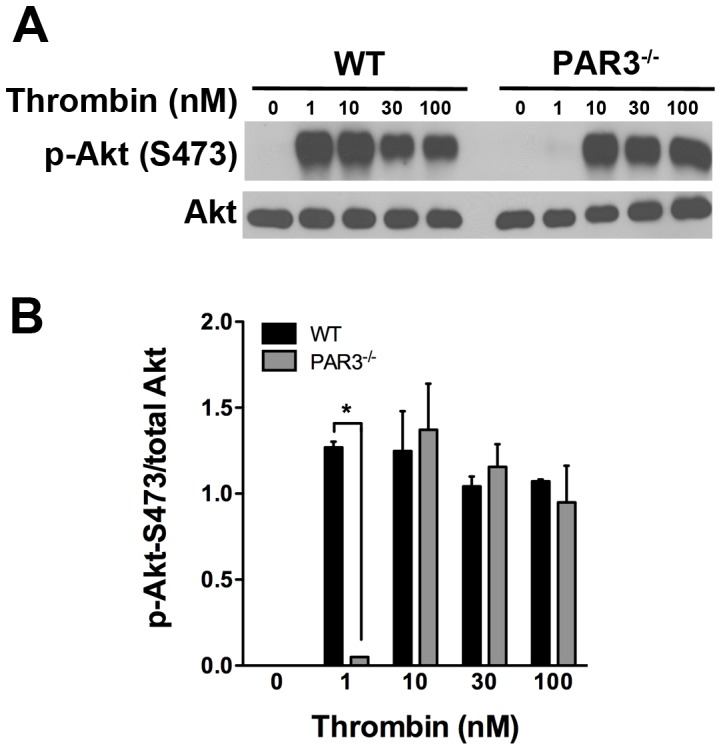
Western blot analysis of Akt phosphorylation in mouse platelets. (**A**) The level of Akt phosphorylation at Ser473 in response to increasing concentrations of thrombin (1–100 nM) was determined by western blotting with phospho-Akt (Ser473) antibody. The membrane was re-probed for total Akt to demonstrate protein loading. The blots shown are from a representative of three independent experiments. (**B**) Quantitation of Akt phosphorylation at (Ser 473) in response to thrombin is represented at the mean (± SD, n = 3) (* *p*<0.05).

PAR4 couples to G_q_ and G_12/13_ in human platelets [7], [8]. We next determined if G_12/13_-mediated signaling was also negatively regulated by PAR3 in response to thrombin in mouse platelets. The G_12/13_ pathway was tested by measuring the activation of the small GTPase RhoA by a G-LISA in response to thrombin. As expected, the level of RhoA activation is decreased in PAR3^−/−^ compared to wild type mouse platelets at low thrombin concentrations (≤10 nM) because PAR4 was unable to mediate the signaling in the absence of PAR3 (Figure 6). However, there was no significant difference in the level of RhoA activation in response to thrombin concentrations (≥30 nM) in PAR3^−/−^ platelets compared to wild type mouse platelets. We next examined the activation of G_i_ pathway in response to thrombin by measuring the phosphorylation level of Akt. The activation of Akt plays an important role in platelet aggregation and secretion by negatively regulating glycogen synthase kinase 3β (GSK-3β) [24], [25]. Our data show that in response to increasing concentrations of thrombin, there was no significant difference in Akt activation between PAR3^−/−^ and wild type mouse platelets (Figure 7A and B). These data indicate that PAR3 negatively regulates PAR4 induced G_q_ signaling pathways without affecting G_12/13_ and G_i_ pathways in mouse platelets.

### PAR3 and PAR4 form constitutive homodimers and heterodimers

**Figure 8:**
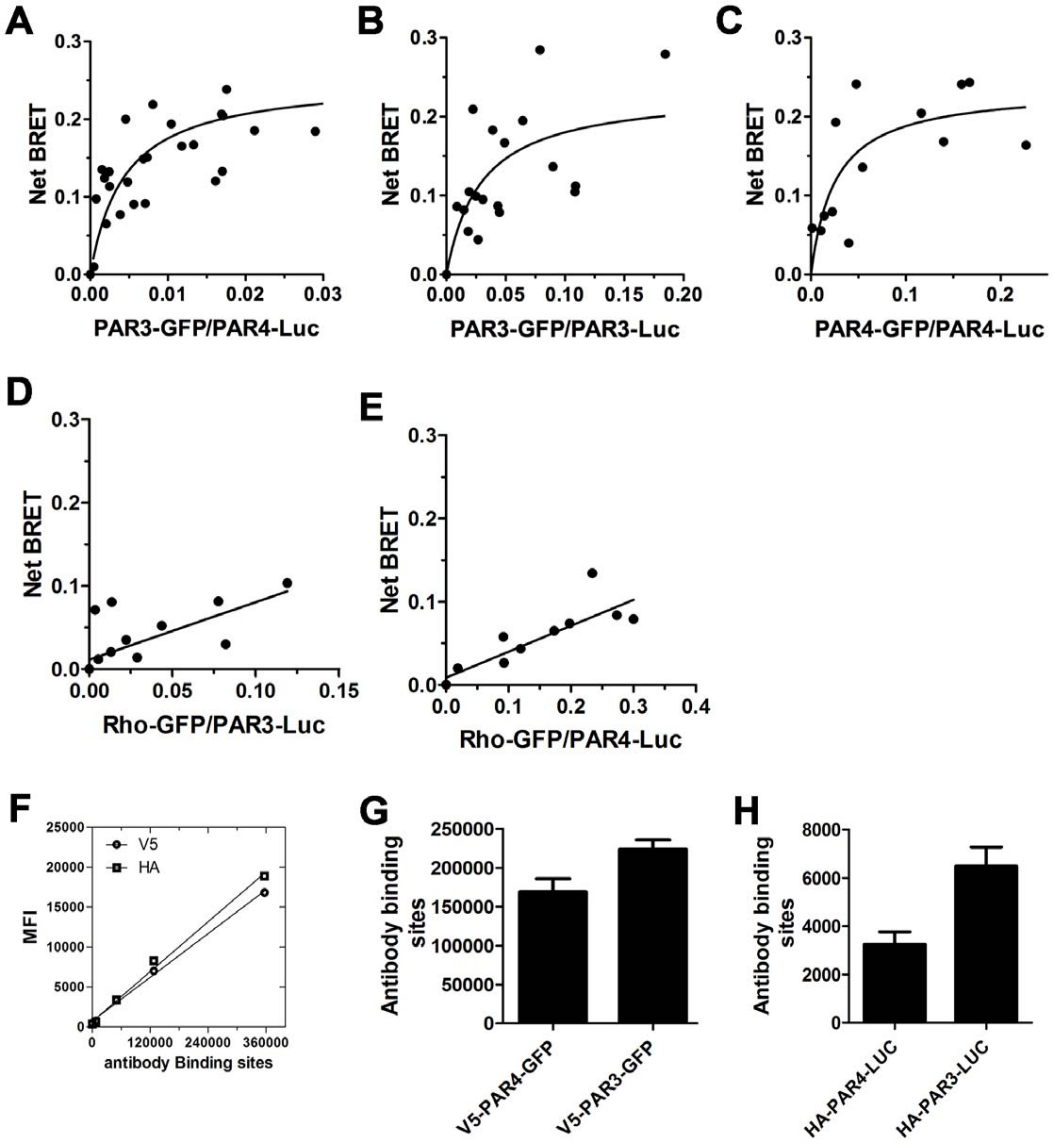
Bioluminescence resonance energy transfer (BRET) analysis of PAR3 homodimers, PAR4 homodimers, and PAR3-PAR4 heterodimers. The HEK293 cells were transfected with: (**A**) PAR4-Luc (1 µg) and PAR3-GFP (0–2.5 µg), (**B**) PAR3-Luc (1 µg) and PAR3-GFP (0–2.5 µg), or (**C**) PAR4-Luc (1 µg) and PAR4-GFP (0–2.5 µg). As a control experiment, the HEK293 cells were transfected with: (**D**) PAR3-Luc (1 µg) and rho-GFP (0–0.12 µg), or (**E**) PAR4-Luc (1 µg) and rho-GFP (0–0.12 µg). Forty-eight hours post-transfection, the cells were analyzed for GFP expression, Luc expression, and BRET. The curves were plotted as the ratio of GFP to Luc and all points from 3–6 independent experiments were analyzed by global fit to a hyperbolic or linear curve. The surface expression of PAR3 and PAR4 in the HEK293 cells was determined by flow cytometry. (**G**) V5-PAR4-GFP and V5-PAR3-GFP were detected with a V5 tag antibody conjugated to Alexa Fluor 647. (**H**) HA-PAR4-LUC and HA-PAR3-LUC were detected with a HA tag antibody conjugated to Alexa Fluor 647. The results are the mean (± SD) of two independent experiments. The number of PAR4 and PAR3 molecules on the HEK293 cells surface is calculated from the V5 or HA antibody standard curve using quantitative flow cytometry (**F**).

To address the mechanism of how down-regulation of mouse PAR3 affects mouse PAR4 signaling, we investigated the possibility that PAR3 and PAR4 physically interact using bioluminescent resonance energy transfer (BRET) [21]. Initial studies examined the PAR3-PAR4 heterodimer (Figure 8A). PAR3 and PAR4 formed heterodimers as indicated by a hyperbolic BRET signal in response to an increase in the PAR3-GFP: PAR4-Luc ratio. We next determined that PAR3 and PAR4 also formed homodimers (Figure 8 B and C) and PAR3 or PAR4 were unable to form heterodimers with rhodopsin (Rho) (Figure 8 D and E). These data demonstrate that PAR3 specifically interact with PAR4 and form constitutive homodimers and heterodimers. Finally, we verified the expression level of PAR3 and PAR4 in HEK293 cells by flow cytometry using HA or V5 tag antibodies conjugated to Alexa Fluor 647. The mean fluorescence intensity from each antibody was converted to antibody binding sites using quantitative flow cytometry (Figure 8 F, G and H).

## Discussion

The accepted physiological role of PAR3 in mouse platelets is to serve as a cofactor for cleavage and activation of PAR4 at low thrombin concentrations [6]. The results from the current study provide the first evidence that PAR3 plays an additional role in mouse platelets by negative regulation of PAR4 mediated Ca^2+^ mobilization and protein kinase C (PKC) activation without affecting the downstream signaling of the G_12/13_ pathways. Throughout our study we have used thrombin concentrations of 30 and 100 nM. It is common to use low thrombin concentrations to examine signaling pathways in platelets so that one can detect subtle differences that would otherwise be missed. It is important to consider that the thrombin concentration generated at the platelet surface at the site of injury likely reaches >100 nM locally [19].

In human platelets, the elevation in intracellular Ca^2+^ concentration regulates various platelet functions, such as integrin activation, granule secretion, and rapid procoagulant phosphatidylserine (PS) exposure [26], [27]. One important initiator of Ca^2+^ signaling is the activation of G_q_ pathways, which induce the generation of diacylglycerol (DAG) and inositol-1,4,5-triphosphate (IP_3_) to activate PKC and Ca^2+^ store depletion, respectively [28]. In platelets, the major Ca^2+^ entry pathway is mediated by Ca^2+^ channels known as store-operated calcium entry (SOCE). The SOCE channels are activated by depletion of intracellular Ca^2+^ stores induced by IP_3_ generated downstream of G_q_ [29]. In this study, we have shown that platelets from PAR3^−/−^ mice have 1.6-fold increase in the maximum intracellular Ca^2+^ mobilization (Figure 1), an increase in phosphorylation level of PKC substrates (Figure 4), and a 2-fold increase in Ca^2+^ release from the stores (Figure 5) in response to thrombin (30–100 nM) or AYPGKF. Our results from Ca^2+^ store depletion are consistent with previous data that show an increase in IP_3_ formation in COS7 cells transfected with PAR4 compared to COS7 transfected with both receptors (PAR4 and PAR3) in response to thrombin (10–100 nM) [6]. It has been shown that PAR1, but not PAR4, negatively regulates intracellular Ca^2+^ mobilization and procoagulant phosphatidylserine (PS) exposure in a PKC-dependent mechanism in human platelets [30]. Our data show that PAR3 negatively regulates Ca^2+^ mobilization and PKC activation in response to high thrombin concentration or PAR4 agonist peptide, perhaps by a physical interaction with PAR4 in mouse platelets. Further, platelets from PAR3^+/−^ had an intermediate increase in Ca^2+^ mobilization (Figure 1A and B). These data support that PAR3 is directly influencing signaling from PAR4. In platelets, PAR4 also interacts with the P2Y_12_ receptor in response to thrombin [23]. Therefore, it is also possible that PAR4 and P2Y_12_ heterodimers are increased in the absence of PAR3, which influences PAR4 mediated increase in the maximum Ca^2+^ mobilization. However, our results show that blocking ADP signaling with 2MeSAMP does not affect the Ca^2+^ mobilization in response to thrombin (30 and 100 nM) or AYPGKF (1.5 and 2 mM) in PAR3^−/−^ platelets. These data confirm that PAR3 is affecting the Ca^2+^ signaling downstream of PAR4 independently of P2Y12.

PAR subtypes communicate with one another to modulate signaling [14], [15], [21], [31]. It has been reported that PAR3 is able to enhance the cleavage of PAR4 with thrombin in cells expressing PAR4 and the N-terminal domain of PAR3 linked to CD8 [6]. It is unlikely that the PAR3-CD8 is dimerizing with PAR4. These data indicate that the interaction between PAR3 and PAR4 is not required for enhanced cleavage of PAR4 by thrombin. Our data suggest that PAR3's ability to enhance PAR4 cleavage is distinct from its influence on PAR4 signaling. We demonstrate in the current study that PAR3 directly interacts with PAR4 and forms constitutive homodimers and heterodimers by using bioluminescent resonance energy transfer (BRET) (Figure 8). The balance between PAR3 homodimers, PAR4 homodimers, and PAR3-PAR4 heterodimers maybe altered in the absence of PAR3 in mouse platelets, which may influence the G_q_ signaling pathway. It is widely accepted that PAR3 does not signal on its own. However, there are two examples of PAR3 regulating signaling from other PAR family members. McLaughlin *et al*. showed that the activation of PAR1-PAR3 heterodimers with thrombin induces distinct signaling from PAR1-PAR1 homodimers [17]. A second example is in podocytes where PAR3 influenced activated protein C (APC)-mediated cytoprotective signaling through PAR1 in mice or PAR2 in humans [18]. The debate between monomers, dimers, and oligomers is ongoing in the GPCR field. However, recent technological advances using sophisticated FRET detection systems have suggested that some GPCRs form parallelogram shaped tetramers [32]. In addition, one report using this technique has demonstrated an excess of dimers in addition to tetramers [33]. The authors hypothesize that there are two interfaces of the complexes with differing affinities, a high affinity site (dimers) and a lower affinity site for the tetramers (a dimer of dimers). It is quite possible that we are seeing dimers of PAR3 interact with dimers of PAR4. As discussed above, PAR4 also forms heterodimers with the P2Y_12_ receptor [23]. The P2Y_12_ receptors may be oligomerizing with dimers of PAR4 in a similar manner. At this point we are limited by the technology to detect these higher order structures in a quantitative manner in native platelet membranes. The understanding of how GPCRs cooperate physically to mediate signaling is crucial to understanding their function and should be the focus of future studies

PAR4 is also coupled to G_12/13_ in platelets [7]. The activation of the G_12/13_ pathway by thrombin induces the activation of the small GTPase RhoA which regulates dense granule release and platelet shape change [7]. Our data show that the activation level of RhoA-GTP (Figure 6) is not affected in PAR3^−/−^ platelets compared to wild type mouse platelets in response to thrombin (30–100 nM). These results demonstrate that PAR4 signaling through G_q_, but not G_12/13_, is regulated by PAR3. The direct coupling of PAR4 to G_i_ in platelets has been attributed to indirect activation of G_i_ pathways via secreted ADP acting on its receptors [12]. In other studies, Akt activation downstream of PARs was G_12/13_ and G_i_ dependent, but independent of G_q_ [8]. In our studies, we have used Akt phosphorylation as a measure of G_i_ activation. There were no significant differences between wild type platelets and PAR3^−/−^ platelets (see Figure 7). There is one report that Akt phosphorylation is downstream of phospholipase C (PLC) in human platelets [34]. Reséndiz *et al*. showed that late Akt phosphorylation was dependent on PLC, calcium, PKC and PI3K in human platelets stimulated with thrombin (1 U/mL) or AYPGKF (0.25 mM). In our study we show significant differences in Ca^2+^ mobilization and PKC activation in response to thrombin concentrations (≥30 nM which corresponds to ≥4 U/mL) or AYPGKF concentrations (≥0.5 mM) in PAR3^−/−^ compared to wild type mouse platelets. However, we do not see changes in Akt phosphorylation in our studies. It is possible that at high agonist concentrations for 3 minutes, the activation of Akt is driven primarily by G_i_ signaling rather than PLC. Taken together, our results show an increase G_q_ dependent signaling in PAR3 ^−/−^ mice.

In summary, when PAR4 is activated in the absence of PAR3 with high concentrations of thrombin (≥10 nM) or PAR4 activated peptide (≥0.5 mM), the G_q_ signaling pathway is increased. In order to explain the increased Ca^2+^ mobilization in PAR3^−/−^ platelets, which affects the Gq-dependent signaling, but not G12/13-dependent signaling, we considered the following hypotheses. First, PAR3 can regulate G_q_ signaling indirectly through PAR4. The absence of PAR3 may induce a conformational change in PAR4, which increases the activity of G_q_. However, PAR4 is also coupled to G_12/13_ and a conformational change in PAR4 would also affect the signaling downstream of G_12/13_. A global change in PAR4 activity by PAR3 is not consistent with our results since the G_12/13_ signaling pathway was not affected in PAR3^−/−^ mice. A second hypothesis is that the expression or distribution of proteins such as RGS (Regulator of G-protein Signaling) is altered in the PAR3^−/−^ mice. A recent study showed that preventing RGS/Spinophilin/and tyrosine phosphatase SHP-1 complex formation in platelet produced a gain in function and increase G_q_-mediated signaling [35]. However, as PAR3^+/−^ platelets produced an intermediate level of Ca^2+^ mobilization, it is unlikely that RGS expression, function, or both are affected in PAR3^−/−^. A third hypothesis is that PAR3 selectively regulates G_q_ signaling by direct contact with PAR4 and this is altered in PAR3^−/−^ mice due to a change in the ratio of PAR4 homodimers to PAR3-PAR4 heterodimers. This hypothesis is supported by the study carried out on the serotonin 5-hydroxytryptamine2C (5-HT2C) receptors one of the largest subfamilies of GPCRs expressed in platelets [36]. The results of this study show that 5-HT2C homodimer interacts with a single G_q_ protein and the dimerization plays a functional role in regulating the activity of 5-HT2C receptors expressed in HEK293 cells. Recent studies from our laboratory have shown that human PAR4 forms constitutive homodimers at the plasma membrane [21]. Mutations at the PAR4-PAR4 homodimer interface disrupted dimer formation and reduced Ca^2+^ mobilization in response to the PAR4 agonist peptide [21]. PAR3-PAR4 heterodimer may have reduced capacity in comparison to the PAR4 homodimer. The increased regulation of signaling due to the physical interaction may allow for fine-tuning of responses from GPCRs. A recent study by McCoy *et al*. was able to differentiate the binding site for G_q_ and G_12/13_ on PAR1 [10]. The specific region on PAR4 that interacts with G-proteins is unknown. Future studies that examine these interactions may also uncover how PAR subtypes modulate PAR4 signaling. These are important considerations and potential consequences for PAR4 interactions with PAR1 on human platelets.

In conclusion, this work reports that PAR3 negatively regulates PAR4-mediated Ca^2+^ mobilization and PKC activation at physiologic thrombin levels by a physical interaction with PAR4 without influencing G_12/13_ signaling. The influence on G_q_ signaling is distinct from PAR3 enhancing PAR4 activation at threshold thrombin levels, as described previously by Nakanishi-Matsui *et al*. [6]. Given the importance of PAR4 activation in platelets of multiple species, the interactions of PAR4 with other receptors may influence downstream signaling and cellular response. Uncovering the details of these interactions is essential to fully understanding the optimal targets for future antiplatelet therapies. These studies also shed light on how PAR3 may modulate signaling in other cell types to modulate specific signaling cascades.
