# Correction: Calcium Mobilization And Protein Kinase C Activation Downstream Of Protease Activated Receptor 4 (PAR4) Is Negatively Regulated By PAR3 In Mouse Platelets

**DOI:** 10.1371/annotation/0956857d-ecf5-4fb7-a834-131915a38a6a

**Published:** 2013-12-09

**Authors:** Amal Arachiche, María de la Fuente, Marvin T. Nieman

There is an error in reference 33. The correct reference is: Patowary S, Alvarez‑Curto E, Xu TR, Holz JD, Oliver JA, Milligan G, Raicu V. (2013). The muscarinic M3 acetylcholine receptor exists as two differently sized complexes at the plasma membrane. Biochem J 452:303-12. 

